# TH Open—Editor's Highlights of 2024

**DOI:** 10.1055/a-2513-4445

**Published:** 2025-01-27

**Authors:** Rory R. Koenen

**Affiliations:** 1 Department of Biochemistry, CARIM School for Cardiovascular Diseases, Maastricht University, Maastricht, The Netherlands

The year 2024 has passed in the blink of an eye, but not without leaving its landmarks inside and outside of the academic world. This also applies to TH Open, which was in the privileged situation to host several excellent contributions from the hemostasis and thrombosis community and continues to be a valuable and visible platform for publications in this field. With the year 2025 ahead, it will be even more worthwhile to publish in TH Open, as the journal is now taken up in the Web of Science Clarivate Emerging Sources Citation Index (ESCI). This means that citations will be indexed, and it also opens the way toward an impact factor.

The end of the year is a good occasion for reflection and the selection of key contributions to TH Open. The year 2024's selections have in common that they all focus on bleeding and its management. For example, the study by Kongebro and colleagues investigated the bleeding risk associated with oral anticoagulant therapy during atrial fibrillation in a group of over 6,000 patients, with a subgroup that was monitored by an implantable loop recorder. 1 The risk for bleeding events increased two-fold after initiation of anticoagulation but was notably lower in the subgroup that was monitored by the loop recorder, and comparable to the risk that accompanied standard care. Interestingly, the risk for bleeding was associated with age, where individuals over 75 were at higher risk for bleeding than those of younger age.

With the development of innovative coagulation factor- and antibody-based therapeutics, hemophilia has become a manageable disorder. Still, people living with hemophilia face several quality-of-life limiting challenges. The article by Chowdary and coworkers has mapped the unmet needs of people with hemophilia (A and B) from the Cost of Haemophilia in Europe: a Socioeconomic Survey-II (CHESS II) and CHESS in the pediatric population (CHESS PAEDs) studies. 2 In the adults from the CHESS II study group, 37% received factor VIII prophylaxis versus 68% in the CHESS PAEDs, and the groups were stratified according to treatment regimen and disease severity. The annual bleeding rates were up to 4.9 in all observed groups and problem joints were observed in 44% of the adult and 23% of the pediatric patients. Chronic pain was reported by a majority of patients (74% of adults and 59% of young). Finally, quality-of-life scores reported by the patients with hemophilia were lower than the reported scores from a comparable group of healthy individuals. Thus, despite the availability of treatment and regardless of disease severity, people with hemophilia are confronted with unmet challenges that need to be addressed, for example, by further optimization of treatment.

The treatment of hemophilia is under continuous development to optimize administration intervals and counteract the detrimental occurrence of inhibitors. A new approach to supplement factor VIII activity is the use of bispecific antibodies such as emicizumab, an antibody that recognizes both factor X and factor IXa, thereby replacing the cofactor function of factor VIII. In the study by Castaman and collaborators, the safety and tolerability of emicizumab were assessed in a phase IIIb multicenter trial in patients with hemophilia (aged >12 years) who underwent surgery. 3 Among the 46 participants included, 56 underwent minor and 22 underwent major surgeries. The majority of participants who underwent major surgeries received additional hemostatic medication (>80%), while more than half of the minor surgeries could be managed without additional medication. Major surgeries had a higher rate of postoperative bleeds, >50% of which required treatment. The most common medication for bleeding management was recombinant factor VIIa. The study concluded that prophylaxis with emicizumab presents a safe and tolerable approach for people suffering from hemophilia undergoing surgeries, with a significant proportion of postoperative bleeds being controllable by bleeding management.

A further highlight focuses on the treatment of hemophilia B and the prophylaxis of bleeding using a recombinant fusion protein linking coagulation factor IX to albumin (rIX-FP, IDELVION, CSL Behring, Marburg, Germany) in 12 previously untreated patients with moderate to severe factor IX deficiency, ranging from 0 to 11 years of age. 4 The treatment lasted at least 50 (exposure) days and did not result in the development of inhibitors in 11 out of 12 patients. Of the 137 adverse events that emerged upon treatment, 5 were attributed to the rIX-FP agent. No thromboembolic events were observed. The authors concluded that the prophylaxis and on-demand treatment with rIX-FP were safe, effective, and well-tolerated.

Finally, an algorithm for the management of urgent bleeding in patients with hemophilia A is outlined, which will aid the non-specialist health care worker to assist in the management of bleeding in hemophilia patients with or without FVIII inhibitors who are under emicizumab prophylaxis. 5

Taken together, the above studies highlight the importance of care and management of bleeding disorders, particularly hemophilia, also taking the challenges of the patients and physicians into account. As the editor-in-chief, I am grateful to the authors for selecting TH Open as a platform for the dissemination of their important findings. I also would like to thank the excellent team of scientific editors and the journal management team for their invaluable work in 2024 and for the efforts to further shape the journal's profile in 2025 and beyond.
